# Cell death proteins in sepsis: key players and modern therapeutic approaches

**DOI:** 10.3389/fimmu.2023.1347401

**Published:** 2024-01-11

**Authors:** Chloe S. Yang, Craig M. Coopersmith, John D. Lyons

**Affiliations:** ^1^ Department of Surgery, Emory University, Atlanta, GA, United States; ^2^ Emory Critical Care Center, Emory University, Atlanta, GA, United States

**Keywords:** sepsis, cell death, apoptosis, necroptosis, pyroptosis, inflammation

## Abstract

Cell death proteins play a central role in host immune signaling during sepsis. These interconnected mechanisms trigger cell demise via apoptosis, necroptosis, and pyroptosis while also driving inflammatory signaling. Targeting cell death mediators with novel therapies may correct the dysregulated inflammation seen during sepsis and improve outcomes for septic patients.

## Introduction

Programmed cell death (PCD) networks are fundamental components of the host response to infection (1, 2). These front-line defense mechanisms both eliminate infected cells and trigger inflammation, and they are increasingly recognized as significant contributors to the dysregulated immune environment of sepsis (3–7). Importantly, these networks display significant overlap and interplay with one another, and attempts at manipulating PCD cascades must contend with diverse and complex signaling outcomes. This review will highlight core mediators of PCD pathways and examine current and potential future therapeutic strategies to target cell death proteins in sepsis.

## Key PCD components

### Caspases

**Figure 1:**
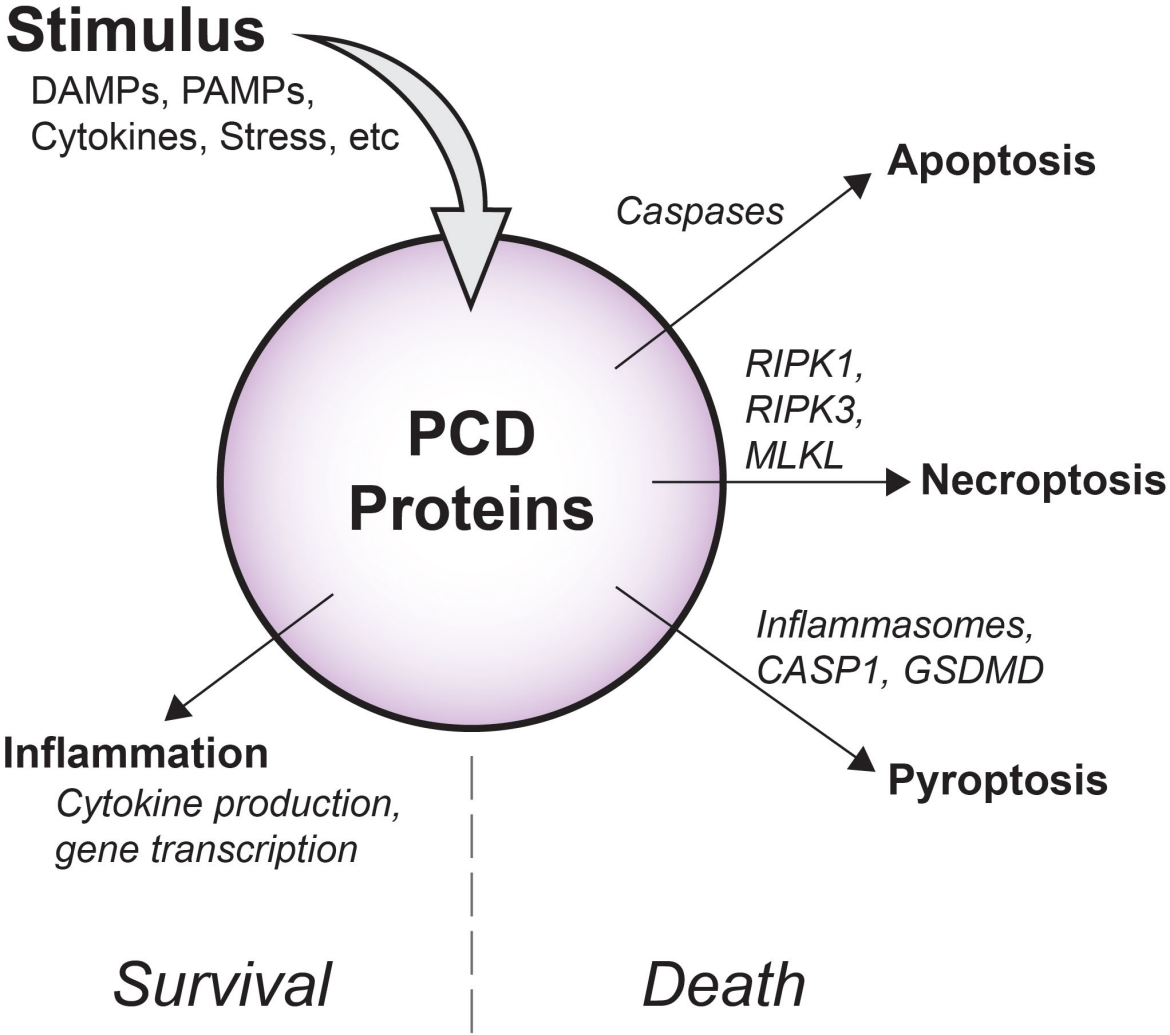
Overview of diverse PCD signaling outcomes. Depending on cellular conditions, flux through PCD signaling pathways has variable outcomes. Apoptotic caspases drive non-inflammatory cell death via apoptosis, while RIPK1, RIPK3, and MLKL support inflammatory membrane rupture via necroptosis. As part of inflammasomes, pro-inflammatory CASP1 promotes cleavage of GSDMD and cell death by pyroptosis. Importantly, in some contexts, these molecules may also induce inflammatory gene transcription and cytokine production that occurs without causing cell death.

The caspases are a family of highly evolutionarily conserved proteases that cleave peptide bonds within proteins by hydrolysis. Perhaps best known as mediators of apoptosis (**Figure 1**), caspases are now understood to also support inflammatory signaling, and distinct groups of pro-inflammatory and apoptotic caspase members have been well described (8, 9).

Inflammatory caspases (also called “Group I caspases”) cleave inactive mediators into their functional forms (9). Caspase-1 (CASP1) was first identified as the enzyme responsible for the conversion of pro-interleukin-1β (IL-1β) to IL-1β, and it plays a similar role in the maturation of IL-18. Group I also includes CASP4 and CASP5. These proteases do not participate in IL-1β production, but rather, in concert with CASP1, hydrolyze and activate gasdermin-D (GSDMD), a pore-forming molecule that perforates cell membranes to release cytokines and damage associated molecular patterns (DAMPs) in the lytic cell death mode of pyroptosis (**Figure 1**). Importantly, CASP4/5 (and the murine equivalent, CASP11), also bind directly to cytosolic lipopolysaccharide (LPS) which causes auto-activation, demonstrating that inflammatory caspases also function as direct pathogen sensors (10).

Apoptotic caspases include both initiator and effector varieties (“Group II” and “Group III” caspases, respectively) (9). When activated, these enzymes cleave hundreds of target sequences within cell proteins, facilitating homeostatic tissue turnover and non-inflammatory cell death by apoptosis (9). Unlike pyroptosis, apoptosis does not disrupt cell membrane integrity, and cell components are broken down and packaged into apoptotic bodies for orderly clearance by phagocytes. A plethora of stimuli promote apoptotic caspase signaling, including mitochondrial stress, DNA damage, and infection (11).

**Figure 2:**
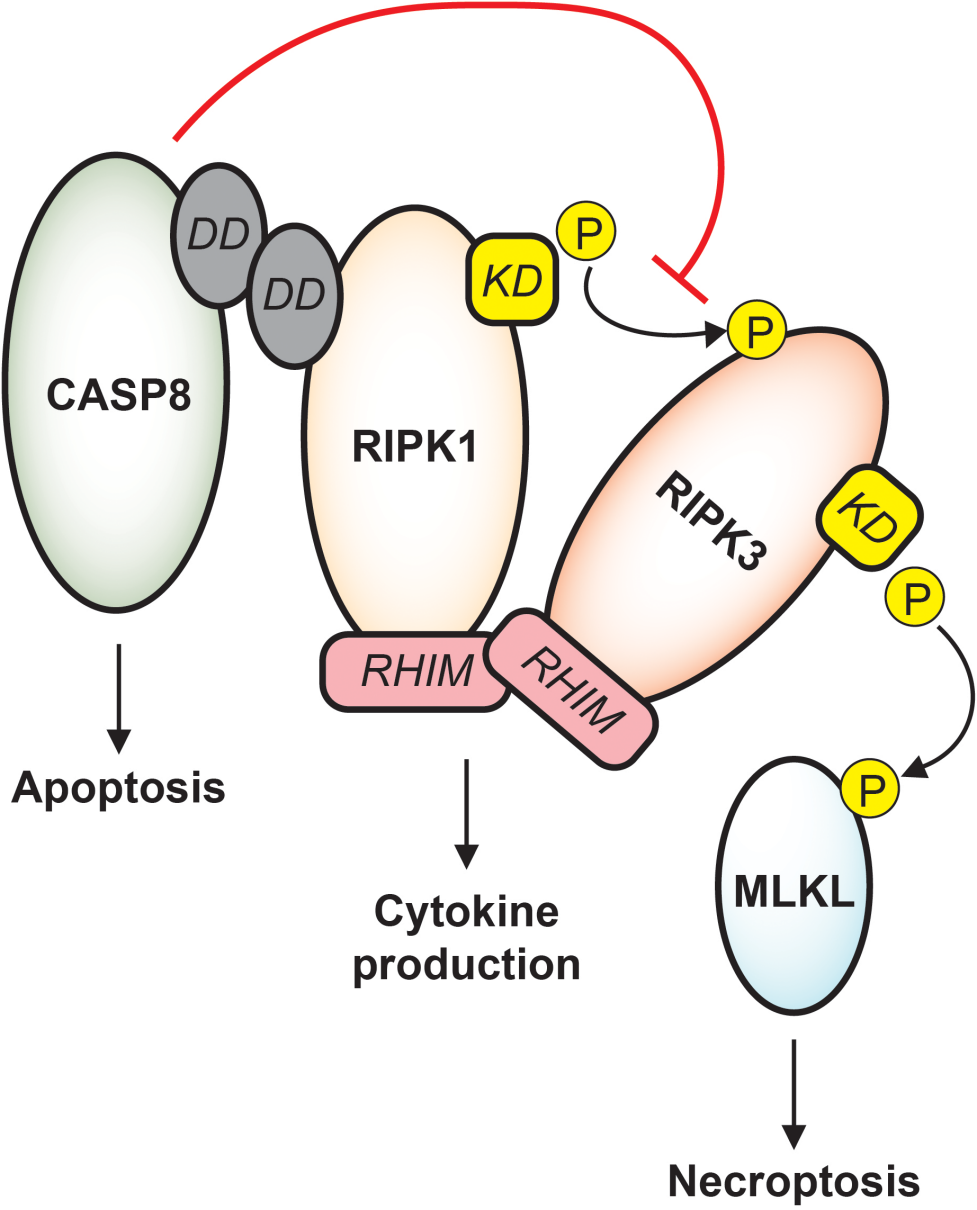
Apoptotic and necroptotic crosstalk. CASP8 interacts with RIPK1 via death domain (DD) binding. Downstream of TNF receptor signaling, CASP8 drives apoptosis while inhibiting necroptosis. RIPK1 and RIPK3 binding is stabilized by RHIM-RHIM interactions, and under conditions of CASP8 inhibition, the RIPK1 kinase domain (KD) phosphorylates RIPK3 which phosphorylates MLKL, causing cell death by necroptosis. RIPK1/RIPK3 RHIM interactions also support cytokine production that is independent of both apoptosis and necroptosis, and destabilization of RIPK1 or RIPK3 may trigger caspase-mediated apoptosis.

Sepsis induces broad and diverse caspase activity, and the resulting impact on septic immune dysfunction is complex and context-dependent. Initial descriptions of apoptotic caspases in sepsis noted pronounced death of immune cells and gut epithelial cells (12), and later studies revealed that transgenic animals with inhibited apoptotic machinery consistently display improved sepsis survival (13–15). Inflammatory caspases support robust cytokine production early in sepsis and may trigger pyroptosis in infected patients, though whether these signaling outcomes are beneficial or detrimental to the host cannot be uniformly stated (16–19). Moreover, it must be emphasized that substantial crosstalk occurs between caspase subfamilies and other cell death machinery, and any therapeutic approach based on caspase modulation must consider complicated signaling dynamics (8). For instance, caspase-8 actively protects cells against stimulation of receptor interacting protein kinase 3 (RIPK3) which would cause cell death by necroptosis, and correspondingly, inhibition of RIPK3 function may predispose cells to death by caspase-mediated apoptosis (**Figure 2**) (20–22). Such trap door mechanisms abound in PCD signaling and must be carefully anticipated to avoid unintended cellular toxicity.

### Receptor interacting protein kinases

The receptor interacting protein (RIP) kinases are a group of ubiquitously expressed proteins that regulate diverse cell functions. Each member of the RIPK family contains a homologous kinase domain and variable structural domains that dictate involvement with a range of cell signaling networks. RIPK1, for example, was first identified by virtue of death domain interactions with the cell surface receptor CD95 and was therefore referred to as “receptor interacting protein”. RIPK1 and RIPK3 are directly relevant to septic immune dysfunction, as each play a central role in mediating cell inflammation as well as death by necroptosis (23).

Necroptosis is a lytic and highly inflammatory mode of cell death and is similar to pyroptosis in its explosive release of intracellular contents (24). However, necroptotic membrane pores are formed by the pseudokinase mixed lineage kinase domain like (MLKL), a protein with general kinase structure that lacks ability to perform phosphorylation (25). Importantly, the two most well characterized upstream activators of MLKL are RIPK1 and RIPK3. In the context of TNF signaling, RIPK1 phosphorylates RIPK3, and RIPK3 in turn phosphorylates MLKL which then oligomerizes into channels that perforate the cell (**Figure 2**) (24). This RIPK1-RIPK3-MLKL sequence is but one example of many paths that lead to necroptosis, some of which proceed directly through RIPK3 and do not require RIPK1 (26). Additionally, activation of upstream regulators does not guarantee RIPK3-mediated cell death. Functional caspase-8 restricts necroptosis, highlighting the interconnectedness of these pathways and emphasizing that ultimate signaling outcomes are highly dependent on cellular conditions (22, 24).

Crucially, RIPK1 and RIPK3 also each facilitate signaling responses that are independent of cell death. The somewhat confusingly titled “death domain” on RIPK1 allows participation in protein complexes that govern signaling outcomes independent of cell death, such as pro-survival cytokine production (23). Furthermore, RIPK1 and RIPK3 each contain a RIP homotypic interaction motif (RHIM), a domain that forms β-amyloid-like binding with other RHIM-expressing proteins. This motif expands the function of these proteins as scaffolds in other death-independent signaling cascades including cytokine production and cell cycle progression (**Figure 2**) (23, 27).

Given the complex array of potential signaling outcomes downstream of RIPK1 and RIPK3, the understanding of their role in sepsis remains incomplete and is actively evolving. Though necroptosis was initially posited as a driver of multiple inflammatory pathologies, including sepsis, evidence of widespread necroptosis in septic humans is lacking, and the hope of limiting septic inflammation by narrowly targeting the RIPK1 or RIPK3 kinase domain is likely misplaced (27, 28). However, cohorts of septic patients do display increased expression of RIPK1, RIPK3 and MLKL (29–31), implying these mediators may be involved in sepsis progression. Additionally, animal data suggests the RIPK3 RHIM is a potent driver of septic inflammation independent of its cell death function (3), and loss of RIPK3, but not MLKL, improves control of viral infection (32). Thus, receptor interacting proteins are likely to contribute to host inflammatory signaling during sepsis, though perhaps via mechanisms that extend beyond necroptosis.

### Inflammasomes

Inflammasomes are cytosolic multi-protein complexes that facilitate inflammatory caspase activity, thus stimulating IL-1β production and pyroptosis (33). Inflammasome formation is based upon recognition of distinct danger signals through various pattern recognition receptors (PRRs). These PRRs bind to distinct pathogen-associated molecular patterns (PAMPs) or DAMPs, thus triggering inflammasome protein oligomerization and the recruitment of an adapter termed “apoptosis-associated speck-like protein containing CARD” (ASC). “CARD” refers to “caspase recruitment domain”, and it is this region of ASC that ultimately allows recruitment and activation of CASP1 (33). Inflammasomes can therefore be viewed as having several variable building block components: families of receptor proteins to detect danger signals, adapter proteins to form signaling complexes, and caspase-1 to cleave downstream targets (34). As mentioned previously, CASP4/5 may function as a direct sensor of LPS and is therefore termed a “non-canonical” inflammasome (35). As with other PCD mediators, the outcome of inflammasome signaling is dependent on cell conditions, and inflammasome activity cannot be assumed to result exclusively in IL-1β production or GSDMD cleavage and subsequent pyroprotis (36).

When activated during infection, inflammasomes represent a key component of the host immune response and are often protective, as loss of inflammasome function worsens bacterial and fungal infections in mice (37, 38). Likewise, in some human sepsis patients, sustained inflammasome activation is associated with improved survival (39). However, this finding is not consistent across sepsis models or patient cohorts, as enhanced inflammasome signaling in some instances correlates with increased mortality (40, 41), and deletion of inflammasome components may actually improve septic animal survival (42). The variability in these data may well reflect the heterogeneity within septic cohorts themselves. Broad analyses of inflammatory activity within septic patient populations have identified both hyper- and hypo-inflammatory endotypes, and these findings support the intuitive hypothesis that it is possible to have both too much and too little inflammation when battling infection (43–45). Thus, while increased inflammasome activity may be detrimental to a hyper-inflammatory endotype, it may prove beneficial in patients with more hypo-inflammatory or immunosuppressed phenotypes. The impact of inflammasome signaling on septic outcomes, as with other PCD mediators, is likely context dependent.

### Nucleic acid sensors

Detection of non-self nucleic acid sequences is a highly conserved form of innate immunity, and PRRs that bind DNA and RNA are intimately involved with host PCD signaling mechanisms (46, 47). Recognition of immunostimulatory nucleic acids by these receptors triggers both cytokine production and host cell death, depending on cell conditions. Circulating levels of DNA and RNA – released either from pathogens or from damaged host nuclei and mitochondria – increase during sepsis, and the impact of this antigen load on the overall immune response during sepsis is increasingly appreciated (48–50).

Nucleic acid sensors with particular relevance to bacterial sepsis include the cGAS-STING pathway, Toll-like receptor (TLR) 9, and z-DNA binding protein 1 (ZBP1). Cyclic GMP-AMP synthase (cGAS) is a sensor found in both the cytoplasm and sub-cellular compartments that binds to DNA and activates an endoplasmic reticulum protein termed “stimulator of interferon genes” (STING) (51). STING activation causes production of Type I interferons and inflammatory mediators and may trigger an array of cell death outcomes including apoptosis, pyroptosis, necroptosis, and autophagy, the breakdown of cellular components mediated by lysosomes (47, 51, 52). TLR9 recognizes unmethylated CpG DNA common in bacterial pathogens to stimulate inflammatory responses (53, 54), while RHIM-containing ZBP1 binds z-form nucleic acid structures and mediates diverse signaling outcomes including cell death (52). Each of these sensors and pathways are now understood to support inflammatory responses during bacterial infections, though how they might contribute to immune dysregulation in overt sepsis remains an open question. One intriguing possibility is that these mechanisms perpetuate a feed-forward cycle of host damage and continuous immune stimulation. In this scenario, PCD signaling causes cell death or damage and release of DNA and RNA molecules, and these are then sensed by PRRs, creating even more inflammation perpetuating host damage. Interestingly, knockout of TLR9 or treatment with circulating DNA scavengers reduces inflammation and improves septic animal survival, suggesting these pathways may indeed contribute to dysregulated immune signaling in sepsis and merit ongoing investigation (48, 55).

## Current and future therapies

### Caspase and RIP kinase inhibitors

Several synthetic caspase inhibitors have been developed with the hope of treating diverse disease processes. Both specific and non-specific inhibitors have been evaluated in clinical trials and have previously been reviewed in detail (56). The pan-caspase inhibitor z-VAD-FMK effectively suppresses broad caspase activity and has been instrumental in detailing necroptotic signaling that arises when CASP8 function is compromised (56, 57). Treatment of septic mice with z-VAD-FMK limits sepsis-induced lymphocyte apoptosis and improves survival (5), as does local thymus injection with another broad caspase inhibitor z-LEHD-FMK (58). More specific CASP1 inhibitors have also shown promise in inflammatory disease models (56). Despite these findings, limited animal data and toxicities in human trials have withheld caspase inhibitors from widespread clinical adoption (56).

Given their emerging role in host inflammatory responses, RIPK1 and RIPK3 have also been targeted with the development of several small molecule kinase inhibitors. Necrostatin-1 (Nec-1) is an allosteric RIPK1 kinase inhibitor that prevents RIPK1-RIPK3 complex formation and inhibits kinase-mediated necroptotic signaling through RIPK1 (59). Though Nec-1 blocks phosphorylation of RIPK3 by RIPK1, it does not block RIPK3 autophosphorylation or necroptotic signaling that proceeds directly through RIPK3-MLKL (60–62). Nec-1 limits inflammation and cell death following LPS challenge (63), but treatment of polymicrobial sepsis in mice with Nec-1 surprisingly resulted in worsened mortality and increased inflammation (64). Such seemingly paradoxical findings have also been documented with use RIPK3 kinase inhibitors. Though pharmaceutical blockade of the RIPK3 kinase domain efficiently restricts necroptosis, it also unleashes caspase-mediated apoptosis, counteracting protective effects of necroptosis inhibition (21, 62). Genetic mutation of the RIPK3 kinase domain may also trigger unintended apoptosis via RHIM signaling, indicating manipulation of these mediators must be approached with care (21). Additionally, it should be emphasized that RIP kinase inhibition would not necessarily limit RHIM-dependent signaling outcomes and as evidenced may actually enhance these signal flux through kinase-independent pathways (3, 4). Ultimately, our limited understanding of the discrete mechanistic contributions of the RIP molecules to septic inflammation limits the therapeutic potential of isolated kinase domain inhibition in sepsis.

### RNA interference

RNA interference (RNAi) is a conserved phenomenon across multicellular organisms whereby specific non-coding nucleic acid molecules inhibit translation of messenger RNA (mRNA) into functional protein, thus silencing gene expression (65). This highly-specific process serves not only as a homeostatic mechanism, but also as an arm of host defense, preventing translation of exogenous genetic material from invading microbes (66). Since its discovery, the concept of RNAi has been harnessed as a therapeutic tool to allow silencing of unwanted gene expression in disease states. This approach often utilizes small interfering RNA (siRNA), which are short, double-stranded RNA sequences that engage native RNAi machinery and suppress expression of the gene of interest (67).

Several siRNA-based therapies have been developed, and at least 10 are either approved or in late-stage clinical trials, treating conditions ranging from amyloidosis to acute kidney injury (68). These agents are not intended to treat sepsis, though preclinical models suggest targeting host PCD machinery may be beneficial, as si-RNA inhibition of BIM, a pro-apoptotic protein, limits lymphocyte apoptosis and improves overall survival in septic mice, and similar results are achieved by repressing CASP8 (69–71). Inhibition of RIPK3 with siRNA limits gut epithelial necroptosis and protects against colitis, an approach that could theoretically limit the gut barrier damage of sepsis and prevent ongoing immune stimulation (72, 73). Additionally, siRNA knockdown of high mobility group box protein 1 (HMGB1), a key DAMP released during necroptosis and pyroptosis (57, 74), rescues mice from septic mortality (75). These data indicate that targeting signal transduction distal to actual cell death events may be a useful strategy to combat PCD-driven inflammation. Despite these promising findings, significant hurdles remain in developing viable siRNA treatments for human septic patients, and no siRNA approaches are being studied in active sepsis clinical trials. Importantly, siRNA therapy would not eliminate existing proteins or prevent them from relaying signals, but rather would only stop new gene expression sometime after administration. Thus, therapies intended to limit early PCD events in sepsis like lymphocyte apoptosis would only be effective if given prior to the septic event (71, 75), and siRNA-mediated inhibition of the initial inflammatory surge of sepsis may not be feasible. Rather, siRNA-based therapies may be better directed at immunosuppressive proteins acting later in the course of sepsis, stopping their production before it occurs. Knowledge regarding the role of PCD proteins in late-phase sepsis is limited, and it remains to be seen if previous success of septic siRNA therapies can be translated to clinical applications.

### Messenger RNA

In contrast to therapies using RNAi, which prevents expression of a given protein, therapies based on messenger RNA (mRNA) delivery induce protein expression by directing mRNA sequences into cells for translation by host ribosomes (76). This approach permits expression of essentially any chosen protein, creating substantial opportunity to modulate cell signaling for therapeutic benefit. Numerous clinical trials evaluating mRNA-based vaccines against viruses and malignancies are actively underway, and mRNA strategies have already proven effective and well tolerated in the widespread use of vaccines against SARS-CoV-2 (77). Considering this success, future mRNA-based sepsis therapies could conceivably be directed at host PCD machinery.

Given their central role in host defense, PCD proteins have been evolutionary targets of invading pathogens seeking to aid infection (78–80). As a result, several microbe-derived molecules that directly inhibit PCD signaling have been identified. *E. coli* expresses a protease that specifically cleaves RHIM domains, limiting RIPK1 and RIPK3-mediated necroptosis, and cytomegalovirus (CMV) infection produces proteins that restrict both RHIM interactions and caspase-8 function (79, 81–83). In theory, these PCD inhibitors could be coded into mRNA and delivered as a therapy. mRNA for the CMV protein M45, a RHIM inhibitor, does reduce cytokine production from infected macrophages *in vitro*, though it is unknown how such an approach would impact the widespread inflammation associated with sepsis (3). mRNA technology is rapidly evolving, with novel refinements being made to improve the efficacy and specificity of delivery, and preclinical animal models demonstrate the feasibility of combating infection with mRNA-based tools (84). Considering exogenous mRNA can be translated into protein within hours of delivery, it seems feasible that deleterious effects of host PCD signaling could be interrupted by timely administration of future mRNA therapies. However, mRNA therapies for sepsis have yet to be evaluated in trials, and ongoing studies will need to more clearly define how possible immunostimulatory effects of mRNA delivery will impact the dysregulated immune state of sepsis.

### CRISPR/Cas systems

CRISPR/Cas technology has revolutionized gene editing and has been extensively reviewed elsewhere (85–87).While the CRISPR-associated (Cas) protein Cas9 cleaves DNA at specific sites to allow permanent gene editing (which would not be a viable approach for human sepsis patients), mutated “dead” Cas9 proteins (dCas9) have been developed that lack enzymatic activity and cannot cut DNA but still bind to target sequences (86). Coupled with promotor or suppressor proteins, dCas9 fusion proteins can function as specific and temporary silencers or inducers of gene expression (86, 88). Cas9 proteins can be delivered as intact, functional proteins, but they can also be delivered in the form of mRNA precursors that are then translated into active dCas9 molecules (89, 90). Though this novel technology is still in its infancy, Cas9 systems have been utilized to suppress inflammasome function in skin disease (90), and it is conceivable that similar approaches could someday be incorporated into clinical trials aiming to fine-tune pathologic PCD signaling in septic patients.

## Conclusion

Programmed cell death mediators and their associated networks are central components of the host immune response during sepsis, dictating cell fate and directing inflammatory cascades. Several PCD mechanisms show promise as therapeutic targets in sepsis, though the substantial interconnectedness within PCD signaling arms requires ongoing analysis and a more complete description before preclinical findings can be translated to clinical use. As our understanding of these pathways increases, novel biotechnologies will offer unprecedented an ability to manipulate elements of PCD machinery and guide inflammatory signaling toward improved outcomes for sepsis patients.

## Author contributions

CY: Investigation, Writing – original draft. CC: Conceptualization, Supervision, Writing – review & editing. JL: Conceptualization, Investigation, Supervision, Visualization, Writing – review & editing.
